# Effect of Sunlight Exposure on Anthocyanin and Non-Anthocyanin Phenolic Levels in Pomegranate Juices by High Resolution Mass Spectrometry Approach

**DOI:** 10.3390/foods9091161

**Published:** 2020-08-23

**Authors:** Vita Di Stefano, Salvatore Scandurra, Antonella Pagliaro, Vincenzo Di Martino, Maria Grazia Melilli

**Affiliations:** 1 Department of Biological, Chemical and Pharmaceutical Sciences and Technologies (STEBICEF), University of Palermo, Via Archirafi 32, 90123 Palermo, Italy; 2 Institute for Agricultural and Forest Systems in the Mediterranean, National Council of Research, Via Empedocle, 58, 95128 Catania, Italy; salvatore.scandurra@cnr.it (S.S.); vincenzo.dimartino@cnr.it (V.D.M.); 3 CREA Research Centre for Cereal and Industrial Crops, 95024 Acireale (Catania), Italy; anto.pagliaro90@gmail.com

**Keywords:** *Punica granatum*, polyphenols, hydrolysable tannins, flavonoids, Ultra High Performance Liquid Chromatography-Orbitrap-Mass Spectrometry

## Abstract

Quali-quantitative analyses of anthocyanins and non-anthocyanin phenolic compounds performed with the use of liquid chromatography coupled with high resolution mass spectrometry, were evaluated in juice of pomegranate fruits (‘Dente di Cavallo’), in relation to different light exposures (North, South, West and East). A total of 16 compounds were identified, including phenolic acids, flavonoids, hydrolysable tannins, and anthocyanins, known for their health-promoting effects. Striking differences were observed about the total phenolic content, which was high in juices from fruits with east- and north-facing position, while it was lower in juices facing south. The greatest contents of total flavonoids and anthocyanins were recorded in fruit juices with southern exposure; however, there are no great differences in the content in phenolic acids. Tannins were mainly synthesized in fruit juices with West exposure. The results showed that the position within the tree had no significant effects on color juice, however, it significantly (*p* < 0.05) affected data on fruit weight, soluble sugars and juice yield. Remarkable synergies existed among polyphenols and phytochemicals in pomegranate juice, but collecting fruits with different solar exposure could enhance different health benefits, i.e., the juices with higher polyphenols content could have more anticancer effect or those with higher tannins content could have more antimicrobial effect.

## 1. Introduction

Pomegranate (*Punica granatum* L.) trees are cultivated worldwide in subtropical and tropical regions. Among the largest countries producers are Iran, India, USA, Turkey, Egypt, Italy, Chile, and Spain [1]. The crop is known since ancient time, for its nutritional, medicinal, and ornamental importance [2].

Spain and Italy are the main European producers. In Italy, pomegranate fruits production amounts to around 60,000 tons per year, and the best producers are the Apulia and Sicily regions. The most cultivated varieties are Dente di Cavallo, Mollar, Acco, and Wonderfull [3].

About 50% of the total weight of the pomegranate is made up of peel and carpellar membranes, important sources of active compounds such as flavonoids; elagitannins; proanthocyanidins; polysaccharides; and minerals such as calcium, magnesium, phosphorus and sodium.

The edible component of the fruit represents approximately 50% of the fruit made up of 80% of arils (fleshy part) and 20% of seeds (woody part). The arils contain 85% water; 10% sugars such as glucose and fructose; 1.5% pectin; organic acids such as ascorbic acid, citric acid, and malic acid; and phenolic compounds such as phenolic acids, flavonoids and anthocyanins. The seeds are instead rich in lipids. The oil that can be extracted is 12–20% of the total weight of the seeds and is characterized by a high concentration of polyunsaturated fatty acids such as linoleic acid and linolenic acid. The seeds also contain proteins, vitamins, fiber, pectins, sugars, and phytoestrogens [4].

The market is constantly growing, which is presumably due to the increasing awareness of consumers of the potential health benefits attributed to the pomegranate trees and their processed products [5,6,7].

Pomegranate juices (PJs) are well known for their beneficial properties, they carry out antioxidant, antimicrobial, anticancer, cholesterol-lowering, anti-atherosclerotic, and anti-diabetic activities [8,9,10,11,12,13,14,15,16]. As anthocyanin-rich food, pomegranate juices have been used at various concentrations to enhance the color, taste and aroma properties; to increase the health-benefits properties; and to improve shelf lives of the fortified foods [17,18,19]. The numerous pharmacological studies associated with the regular consumption of PJs in the prevention of certain diseases and the improvement of health conditions, have made it possible to define the fruit as a functional food. Anthocyanins, ellagic acid derivatives, and hydrolysable tannins were detected in pomegranate juices as responsible for antioxidant activity. Anthocyanins are important flower and fruit pigments; they attract pollinators and seed dispersers and protect plant tissues from photo-inhibition and oxidation resulting from photosynthesis [20].

**Figure 1:**
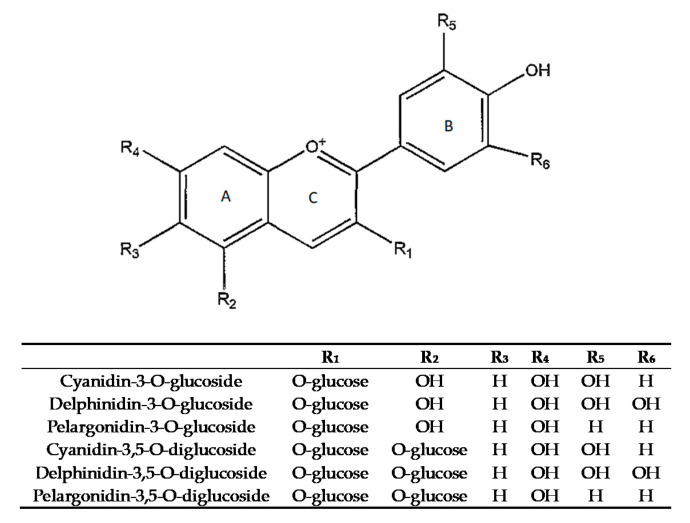
Anthocyanins in pomegranate juices [23].

Anthocyanins act as free radical scavengers, thanks to the *o*-diphenol substitution in ring B of anthocyanins (Figure 1) and the conjugated double-bond system, stabilizing radicals due to hydrogen donation. Metal chelation [21] or DNA and protein binding [22] are important in biological systems.

Delphinidin, cyanidin and pelargonidin glycosides are the most representative anthocyanins in PJs with strong antioxidant activities [23,24,25,26].

As is known, plants and their fruits exhibit physiological responses to solar radiation [27,28]. It is believed that exposure to solar radiation stimulates the production of phenolic substances, which absorb UV rays, to protect tissues from DNA-induced damage [29,30,31].

Direct exposure to sunlight has been shown to regulate the biosynthesis of phenolic compounds by altering the expression of genes that code for enzymes of the biosynthetic pathways [32,33,34,35].

On some types of fruit, it has been observed that the content of anthocyanins and quercetin is far superior in the parts exposed to light, while for other important compounds such as catechins, phloridzin and chlorogenic acid, there are no differences between the fruits exposed to light and those exposed to shade [36].

This work aims to enhance the understanding of the relationship between light of solar radiation on the accumulation of total phenolic content, flavonoids and hydrolysable tannins during fruit ripening.

Pomegranate fruits cv “Dente di Cavallo”, of an experimental orchard located in south-eastern Sicily, Italy, under natural sun irradiance, with North, South, East, and West exposure (Noto N, Noto S, Noto E and Noto W) were collected and total phenolic contents, total anthocyanin contents, and the levels of hydrolysable tannins in the pomegranate juices were determined.

The ultra-high-performance liquid chromatography and high-resolution quadrupole Orbitrap mass spectrometry (UHPLC–Orbitrap-MS) approach was employed to perform phytochemical analyses in PJs of the fruits growing with different light exposure; moreover, color and physicochemical analysis were carried out.

## 2. Materials and Methods

### 2.1. Plant Material, Harvesting Procedures, Preparation of Pomegranate Juices (PJs) and Instrumental Color Measurement

The experimental analysis was conducted on samples of fruits belonging to the cv “Dente di Cavallo”, one of the main cultivars cropped in Sicily. The selected plant material were harvested during November 2018 at the field located in Noto (c.da Fiumara, 36.90° N; 15.05° E, Syracuse, Sicily).

The orchard was established in 1993; hence, trees are 25 years old, it is 2.5 ha. Pomegranate trees were planted at a spacing of 4 m × 4 m. They are drip irrigated, and standard cultural practices are performed (pruning, thinning, fertilization and pest control treatments). Ten trees have been sampled and for each tree, five fruits per four geographical orientation of tree (North-N, South-S, East-E and West-W respectively). Each tree is a biological repetition.

The harvest was performed according the ripening stage, about 180 days after full bloom. Samples were taken from 12:00 h to 14:30 h.

After picking, fruits were immediately transported to the laboratory. Each fruit was carefully cut at the equatorial zone with a sharpened knife, and then intact arils were manually obtained from whole fruits, and the juices were obtained by squeezing them by mechanical press and stored at −20 °C. Weight of the fruits (1 mg accuracy on a balance), weight of the arils per fruit, juice yield (%) and the color of juices (L *, a *, b *) by colorimeter Minolta CR 400 (Konica Minolta, Milan, Italy) were determined [23,37].

### 2.2. Total Phenolic Content (TPC)

Phenolic contents of PJs were determined by Folin–Ciocalteu’s method, and total polyphenols content (TPC) was expressed as grams gallic acid equivalents (GAE) per L^−1^ of juice [23].

### 2.3. Determination of Phenolic Compounds by UHPLC–Orbitrap-MS

Identification of polyphenols (phenolic acids, flavonoids, tannins and anthocyanin-derived pigments) in PJs was based on a reported procedure and the UHPLC–Orbitrap-MS method previously developed [23].

The MS detection was conducted in two acquisition modes: full scan (positive and negative ion modes) and targeted selected ion monitoring. For targeted selected ion monitoring analyses, a mass inclusion list containing exact masses and expected retention times of target phenolic acids, flavonoids, tannins, and anthocyanins analytes was built and applied (Supplementary materials Figures S1 and S3). Phenolic compounds were also identified by MS/MS data-dependent dd-ms^2^ scanning mode.

### 2.4. Statistical Analysis

Data were submitted to the Bartlett’s test and then analyzed using analysis of variance (ANOVA). Means were statistically separated on the basis of Student-Newmann-Kewls test.

## 3. Results

Single fruit weight resulted in being 297 ± 10.97 g, with variations due to solar radiation, influencing the weight of arils and the juice yield (Table 1). Soluble sugars resulted on average 13.4 ± 0.4 °Brix, picked in 14.8 ± 0.77 °Brix in fruit collected in W and S exposure. The color of juice did not show statistical differences in relation to the solar radiation.

**Figure 2:**
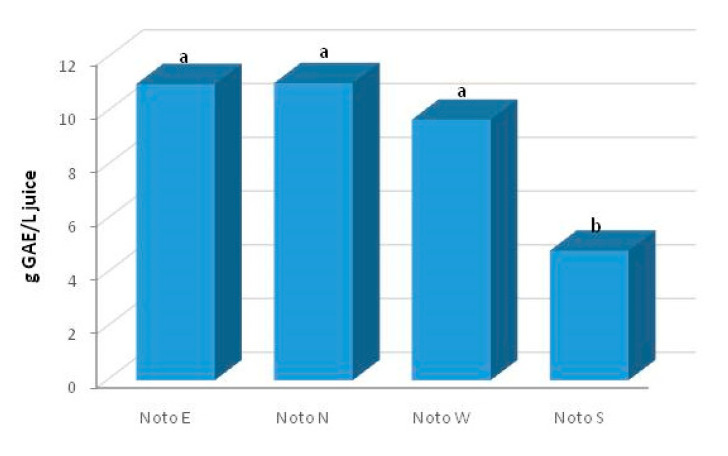
TPC (g GAE L^−1^ juice) of the samples. Different letters indicate differences at *p* < 0.05; *n* = 5.

Total phenolic content (TPC) is an important quality parameter of PJs above all for the organoleptic characteristics, especially color and taste properties and, of course, for the high antioxidant activity. Light exposure during growth greatly affect phenolics accumulation. Phenolic compounds are important secondary metabolites with antioxidant capacity in fruit and were found to be associated with resistance to scald development in fruits [38]. The Folin–Ciocalteu test indicated a high TPC in all samples of juice analyzed. TPC levels ranged from 4.82 g GAE L^−1^ to 11.06 g GAE L^−1^, with the highest amount in Noto N (11.06 ± 0.37 g GAE L^−1^), followed by Noto E (11.02 ± 0.10 g GAE L^−1^) (Figure 2).

Elagitannins, such as punicalagins and punicalins, are the main hydrolysable tannins found in PJs, while, among anthocyanins, cyanidin, pelargonidin, and delphinidin, glucosides are the main bio-molecules characterizing the PJs. The factor “position within the tree” has been studied, besides the differences in TPC, also for the evaluation of the profile in polyphenols in juices.

Identification of polyphenols phenolic acids, flavonoids, and hydrolysable tannins in PJs have been built on reported UHPLC–Orbitrap-MS approach [23].

The presented data in Table 2 show the average values of the fruit juices in the four positions studied (East, West, South and North exposure), for anthocyanins content.

The anthocyanin profile was determined for PJs of different samples, and the chemical structures were determined by the mass spectra in positive ionization mode at different fragmentation energy, and by comparison with literature data.

An inclusion list (Supplementary Materials, Figure S1), in which were reported the molecular formula together with the accurate mass of anthocyanins known, was prepared [23].

Six anthocyanins with a retention time of between 12.20 min and 14.06 min were identified in all the PJs samples. The two available standards were cyanidin-3-O-glucoside and cyanidin-3,5-O-diglucoside. Their use was useful to recognize their presence in the juices analyzed by comparing the retention times. Furthermore, the exact mass and the study of their MS/MS fragmentation was crucial for the identification of the glycosylated anthocyanins (Supplementary Materials, Figure S2).

The quantitative analysis of anthocyanins performed with the use of liquid chromatography coupled with high resolution mass spectrometry, combines the quadrupole selection of the precursor ion with the Orbitrap detection at high resolution and mass precision.

The results proved that the juices studied had the same phenolic qualitative profile but their relative abundances were considerably different. For their quantification, cyanidin 3-O-glucoside *m*/*z* 449.1078 [M]^+^ was the reference compound used for quantification of pelargonidin-3-O-glucoside, delphinidin-3-O-glucoside (Table 3). Cyanidin 3,5-O-diglucoside *m*/*z* 611.1606 [M]^+^ is the reference compound in positive mode for pelargonidin-3,5-O-diglucoside and delphinidin-3,5-O-diglucoside (Supplementary Materials, Table S1).

In particular, Noto S shows the highest content of total anthocyanins (Table 3), followed by Noto N, Noto W and Noto E. Delphinidin, cyanidin and pelargonidin 3-glucosides and 3,5-diglucosides, which are responsible for the red–purple colour of juices, represent the main anthocyanins of the PJs. In decreasing order, the concentrations of anthocyanins in juices were: cyanidin-3,5-O-diglucoside in a range of 305.63–765.69 mg L^−1^; delphinidin-3,5-O diglucoside, 69.43–225.98 mg L^−1^; pelargonidin-3,5-O-diglucoside, 24.52–56.02 mg L^−1^; cyanidin-3-O-glucoside, 69.85–169.74 mg L^−1^; pelargonidin-3-O-glucoside, 8.61–21.66 mg L^−1^; and delphinidin-3-O-diglucoside, 19.66–45.83 mg L^−1^.

In the current study, the levels of anthocyanins obtained are greater than those reported by literature for other cultivar grown in Sicily [39]. In addition, great differences were observed in function of sunlight exposure; Noto S showed higher anthocyanins content (1284.92 mg L^−1^) followed by Noto N (1107.25 mg L^−1^).

An inclusion list was prepared before UHPLC-MS analysis in negative mode of Noto S, Noto N, Noto E, and Noto W samples (Supplementary Materials, Figure S3).

Mass spectra in Full MS, accurate mass and the spectra in MS/MS made it possible to structurally recognize phenolic compounds with retention times between 8.52 min and 20.67 min. Ten compounds have been identified belonging to the class of phenolic acids, flavonoids and tannins, already known in the literature for pomegranate [40].

Figure S4 shows the retention times of the compounds with exact mass collected in the inclusion list. Table 4 shows the tannins, phenolic acids and flavonoids identified in PJ samples in negative mode.

Rutin, gallic acid and cyanidin 3-O-glucoside *m*/*z* 447.093 [M-2H]^−^ were used as reference standards. Cyanidin 3-O-glucoside in negative mode was useful for quantification of vanillic acid hexoside, ferulic acid hexoside, ellagic acid pentoside, ellagic acid deoxyhexoside, kaempferol 3-O glucoside, quercetin 3-O-hexoside, corilagin, and lagerstannin C.

Each phenolic compound was quantified using its respective reference compound of the calibration curves [23]; linear equations and linear regression coefficient of the standards used are reported in the Supplementary Materials (Table S1).

Table 5 shows the quantities of total and individual phenolic compounds expressed as mg L^−1^ contained in the different pomegranate juices.

The compounds are listed below according to their chemical class, and the concentrations are expressed in mg L^−1^ of juice. Among the hydrolysable tannins, similar levels of lagerstannin C and corilagin (10.03–22.01 mg L^−1^ and 6.86–2.66 mg L^−1^, respectively) were found in all samples. Noto W sample has shown a higher content of ellagic acids derivative (29.94 mg L^−1^) and overall a higher concentration of tannins.

The highest concentration of flavonoids was found in the juices of the Noto N and Noto S samples (138.21 and 169.48 mg L^−1^ respectively). Noto N and Noto E had a higher content of phenolic acids hexoside (130.31 and 128.10 mg L^−1^, respectively).

**Figure 3:**
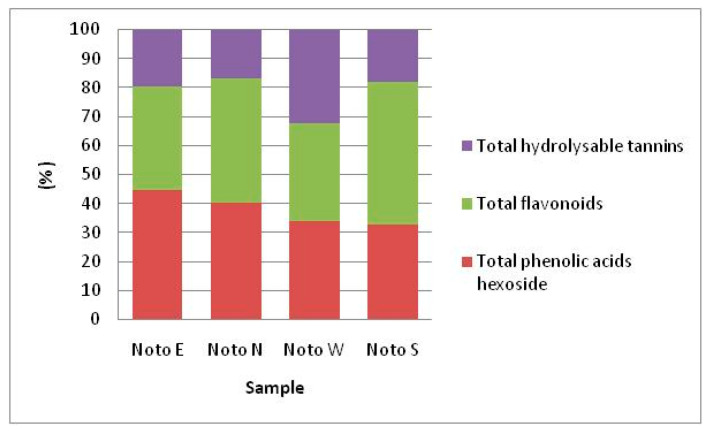
Partitioning (% of total) of the main phenolic compounds in pomegranate juices in relation to solar exposure.

In the present study, pomegranate fruits with South and North exposure had juices with significantly higher concentrations of anthocyanins, Noto S (1284.92 mg L^−1^), followed by Noto N (1107.25 mg L^−1^). Noto S showed the higher content in flavonoids (169.48 mg L^−1^). Noto N with 130.31 mg L^−1^ shower the highest concentration of total phenolic acids hexoside. The relative distribution of each class of phenolic compounds is reported in Table 3. Noto E synthesized mainly total hydrolysable tannins followed by Noto N. Noto S accumulated mainly total flavonoids (Figure 3).

## 4. Discussion

The polyphenol composition of PJs has been characterized as being complex and unique, comprising mainly anthocyanins, phenolic acids and hydrolysable tannins. In this work, we used UHPLC-Orbitrap-MS method for the analysis of both. The main hydrolysable tannins, flavonoids, phenolic acids and anthocyanins in juices of fruits with different sunlight exposures, in order to provide a simple and easily applicable method for quality control of the juices, were identified. Juices of all the samples present similar HPLC-MS profiles, with great difference mainly regarding some anthocyanins contents. The values of TPC recorded in PJs are greater than those reported in Sicilian pomegranate juices by Todaro et al. [39], which ranged from 0.95 to 3.10 g GAE L^−1^, but are similar to the other data reported in literature [41,42]. Differences in reported total phenolic compounds content could be partially explained by pomegranate variety, different water content, environmental growing conditions or ripening stage [43], and pre- and post-harvest treatments [44].

Phenolic compounds are important secondary metabolites with antioxidant capacity associated with resistance to the development of sunburn on the fruit surface [45]. It has been reported that the activity of enzymes involved in the phenolic metabolism pathway can be photonically upregulated [46]. The photoprotective function of anthocyanins has been well documented [47].

Great differences were observed in function of sunlight exposure; PJ with South exposure showed higher anthocyanins content followed by North exposure fruit juices. The highest concentration of flavonoids was found in the juices of the Noto S sample, with a relative percentage of 49.3% of total phenolic compounds. Noto N showed the highest content of phenolic acids hexoside (44.6%), while fruit exposed to West showed 32% in tannins against values lower than 18% in the other samples. Fruit exposure to sun radiation in S, E and W positions might reduce the concentration of flavonoids and phenolic compounds due to elevated temperature, which causes a reduction in their synthesis or even partial degradation. Anthocyanins are typical potent antioxidants and may contribute to hydrogen peroxide scavenging during direct light sun exposure [46]. Based on the markedly higher anthocyanin contents in sun-exposed pomegranate fruits (Noto S) than in the shaded one, we suggest that they might be the main phenolic compounds responsible for protecting fruit from UV/high light damage, where as other class of phenolics are involved to a lesser extent in photoprotection of fruits.

## 5. Conclusions

The obtained results provided evidence that relevant differences on the patterns of anthocyanins in pomegranate fruits can be explained by the effect of light exposure.

Enhanced knowledge of the role played by sunlight intensity on the accumulation of phenolic compounds enables support of cultural practices for a given phenolic profile in pomegranate fruits and fosters the production of high-quality juices.

This study gave new information about the different compositions in bio-molecules with health benefit of PJs in relation to solar exposure of the pomegranate fruits.

## Figures and Tables

**Table 1:** Fruit weight, juice yield, soluble sugars, and juice color coordinates.

	Fruit Weight (g)	Arils Weight (g Fruit^−1^)	Juice Yield (%)	Soluble Sugar (°Brix)	L *	a *	b *
Noto E	276 ± 13.25 ^b^	170 ± 2.04 ^b^	56.9 ± 1.19 ^b^	13.2 ± 0.16 ^b^	40.7 ± 2.12	11.5 ± 0.37	21.88 ± 0.70
Noto N	319 ± 13.40 ^a^	189 ± 4.54 ^ab^	61.5 ± 2.58 ^a^	13.7 ± 0.33 ^b^	40.6 ± 0.97	11.4 ± 0.48	21.75 ± 0.83
Noto W	282 ± 9.02 ^b^	162 ± 8.26 ^b^	63.2 ± 1.52 ^a^	14.7 ± 0.35 ^a^	41.2 ± 1.77	11.7 ± 0.47	21.85 ± 0.59
Noto S	342 ± 8.21 ^a^	218 ± 6.10 ^a^	66.7 ± 3.47 ^a^	14.8 ± 0.77 ^a^	41.3 ±1.16	11.9 ± 0.44	21.7 ± 1.24
Means	297 ± 10.97	179 ± 5.24	59.2 ± 2.19	13.4 ± 0.40	40.9 ± 1.50	11.6 ± 0.44	21.8 ± 0.84

Different letters within the same column indicate differences at *p* < 0.05. All values are mean ± SD of five independent measurements of each sample.

**Table 2:** Anthocyanins content mg L^−1^ of juice of different fruits (SD).

Phenolic Compounds	Noto E	Noto N	Noto W	Noto S
Cyanidin-3-O-glucoside *	69.85 ± 1.06 ^cd^	145.81 ± 1.77 ^b^	87.47 ± 0.39 ^d^	169.74 ± 0.25 ^a^
Delphinidin-3-O-glucoside	21.08 ± 0.19 ^cd^	41.93 ± 0.19 ^b^	19.66 ± 0.23 ^d^	45.83 ± 0.06 ^a^
Pelargonidin-3-O-glucoside	8.61 ± 0.08 ^d^	17.41 ± 0.10 ^b^	9.91 ± 0.03 ^c^	21.66 ± 0.03 ^a^
Pelargonidin-3,5-O-diglucoside	36.87 ± 0.22 ^c^	50.21 ± 0.17 ^b^	24.52 ± 0.02 ^d^	56.02 ± 0.11 ^a^
Cyanidin-3,5-O-diglucoside *	374.00 ± 1.97 ^c^	639.78 ± 1.02 ^b^	305.63 ± 0.14 ^d^	765.69 ± 0.51 ^a^
Delphinidin-3,5-O-diglucoside	118.66 ± 1.42 ^c^	212.11 ± 0.21 ^b^	69.43 ± 0.27 ^d^	225.98 ± 0.14 ^a^
Total anthocyanins	510.41	1107.25	516.62	1284.92

Different letters in row indicate differences at *p* < 0.05. * Commercial standard used for their quantification.

**Table 3:** Identified anthocyanins in positive ionization in pomegranate juices by UHPLC-Orbitrap-MS approach.

Compounds	[M]^+^ *m*/*z*	MS/MS *m*/*z*	Molecular Formula	Retention Time (min)
Pelargonidin-3-O-glucoside	433.1129	271.0593	C_21_H_21_O_10_	14.08
Cyanidin-3-O-glucoside *	449.1078	287.0542	C_21_H_21_O_11_	13.56
Delphinidin-3-O-glucoside	465.1028	303.0492	C_21_H_21_O_12_	13.17
Pelargonidin-3,5-O-diglucoside	595.1658	433.1120271.0594	C_27_H_31_O_15_	13.44
Cyanidin-3,5-O-diglucoside *	611.1606	449.1018287.0542	C_27_H_31_O_16_	12.79
Delphinidin-3,5-O-diglucoside	627.1556	465.1018303.0492	C_27_H_31_O_17_	12.20

MS/MS = Fragmented anthocyanidin molecular weight. This fragment ion is cleavage product free from the sugar moiety. * Commercial standard used for their quantification.

**Table 4:** Phenolic compounds identified in negative ionization in PJs by UHPLC-Orbitrap-MS approach.

Compounds	[M-H]^−^ *m*/*z*	MS/MS *m*/*z*	Molecular Formula	Retention Time (min)
Gallic acid *	169.01344	125.02334	C_7_H_6_O_5_	11.12
Kaempferol-3-O-glucoside	447.09414	285.00387	C_21_H_20_O_11_	13.56
Quercetin 3-O-hexoside	463.08932	300.9990	C_21_H_20_O_12_	13.16
Rutin *	609.14805	447.0571284.9679	C_27_H_30_O_16_	12.87
Vanillic acid hexoside	329.08895	167.03417101.02327	C_14_H_18_O_9_	13.09
Ferulic acid hexoside	355.10476	175.03937160.0130	C_16_H_20_O_9_	14.25
Ellagic acid pentoside	433.04277	300.9990	C_19_H_14_O_12_	19.85
Ellagic acid deoxyhexoside	447.05824	300.9990	C_20_H_16_O_12_	20.67
Corilagin	633.07469	470.98416	C_27_H_22_O_18_	14.20
Lagerstannin C	649.07055	486.97901	C_27_H_22_O_19_	8.52

* Commercial reference compounds used for quantification of individual phenolic constituents.

**Table 5:** Phenolic content expressed as mg L^−1^ of PJs.

Phenolic Compounds	Noto E	Noto N	Noto W	Noto S
Gallic acid *	1.12 ± 0.00 ^b^	0.9 ± 0.04 ^b^	1.21 ± 0.03 ^b^	0.94 ± 0.05 ^b^
Kaempferol-3-O-glucoside	74.94 ± 0.09 ^c^	103.59 ± 0.19 ^b^	62.99 ± 0.11 ^d^	130.80 ± 0.16 ^a^
Quercetin 3-O-hexoside	26.60 ± 0.06 ^c^	33.60 ± 0.03 ^b^	16.96 ± 0.02 ^ef^	37.55 ± 0.04 ^a^
Rutin *	0.74 ± 0.02 ^b^	1.02 ± 0.00 ^a^	0.77 ± 0.00 ^b^	1.13 ± 0.01 ^a^
Vanillic acid hexoside	67.75 ± 0.12 ^b^	70.34 ± 0.01 ^a^	47.52 ± 0.07 ^e^	58.00 ± 0.01 ^c^
Ferulic acid hexoside	60.35 ± 0.01 ^a^	59.97 ± 0.04 ^a^	33.93 ± 0.10 ^c^	53.77 ± 0.26 ^b^
Ellagic acid pentoside	10.48 ± 0.26 ^b^	10.60 ± 0.04 ^b^	13.18 ± 0.08 ^a^	12.36 ± 0.08 ^a^
Ellagic acid deossihexoside	26.64 ± 0.09 ^c^	23.77 ± 0.03 ^d^	29.94 ± 0.06 ^a^	28.32 ± 0.04 ^b^
Corilagin	8.70 ± 0.17 ^b^	6.86 ± 0.02 ^c^	12.66 ± 0.03 ^a^	7.90 ± 0.02 ^bc^
Lagerstannin C	10.03 ± 0.05 ^d^	12.66 ± 0.04 ^c^	22.01 ± 0.06 ^a^	13.01 ± 0.06 ^c^
Total phenolic acids	1.12	0.90	1.21	0.94
Total phenolic acids hexoside	128.10	130.31	81.45	111.77
Total flavonoids	102.28	138.21	80.72	169.48
Total hydrolysable tannins	55.85	53.89	77.79	61.59

Different letters in row indicate differences at *p* < 0.05. * Commercial reference compounds used for quantification of individual phenolic constituents.

## Supplementary Materials

The following are available online at https://www.mdpi.com/2304-8158/9/9/1161/s1, Figure S1: Inclusion list with molecular formula and accurate mass of anthocyanins in positive ionization, by UHPLC–Orbitrap-MS; Figure S2: Retention time (min) of anthocyanins identified in PJs; Figure S3: Inclusion list of phenolic acids, flavonoids and tannins with molecular formula and accurate mass of anthocyanins in negative ionization, by UHPLC–Orbitrap-MS; Figure S4: Retention time (min) of phenolic acids, flavonoids and tannins identified in PJs; Table S1: *m*/*z*, molecular formula, linear regression model and coefficient of determination of external standards used for calibration.
