# Supplementary material for: Effect of Sunlight Exposure on Anthocyanin and Non-Anthocyanin Phenolic Levels in Pomegranate Juices by High Resolution Mass Spectrometry Approach
Source: Foods. 2020 Aug 23;9(9):1161. doi: 10.3390/foods9091161 (PMC7555681; doi:10.3390/foods9091161)

Table S1: *m/z*, molecolar formula, linear regression model and coefficient of determination of external standards used for calibration.

Standard compounds	*m/z*	Molecolar formula		R^2
Gallic acid	169.01344 $[M-H]^-$	$C_7H_6O_5$	$Y = 1.112\ 10^8 \times x$	0.9992
Rutin	609.14805 $[M-H]^-$	$C_{27}H_{30}O_{16}$	$Y = 2.5720\ 10^7 \times x$	0.9669
Cyanidin-3-O-glucoside	447.093 $[M-2H]^-$	$C_{21}H_{21}O_{11}$	$Y = -292208+188967 \times x$	0.9991
Cyanidin-3-O-glucoside	449.10699 $[M]^+$	$C_{21}H_{21}O_{11}$	$Y = 174092+100426 \times x$	0.9915
Cyanidin-3,5-O-diglucoside	611.1589 $[M]^+$	$C_{27}H_{31}O_{16}$	$Y = 561671+170477 \times x$	0.9923

Figure S1: inclusion list with molecular formula and accurate mass of anthocyanins in positive ionization, by UHPLC–Orbitrap-MS.

Method editor — Inclusion List

File Edit Help Done

	Mass [m/z]	Formula [M]	Species	CS [z]	Polarity	Start [min]	End [min]	(N)CE	MSX ID	Comment
1	433.11292	C21H20O10	+ H	1	Positive	8.00	18.00			Pelargonidin-3-O-Glucoside
2	449.10784	C21H20O11	+ H	1	Positive	8.00	18.00			Cianidin-3-O-Glucoside
3	465.10275	C21H20O12	+ H	1	Positive	8.00	18.00			Delphinidin-3-O-Glucoside
4	595.16575	C27H30O15	+ H	1	Positive	8.00	18.00			Pelargonidin-3,5-Di-Glucoside
5	611.16066	C27H30O16	+ H	1	Positive	8.00	18.00			Cianidina-3,5-O-Di-Glucoside
6	627.15558	C27H30O17	+ H	1	Positive	8.00	18.00			Delphinidin-3,5-O-Di-Glucoside
7	737.17123	C36H32O17	+ H	1	Positive	8.00	18.00			Afzelechin-Delphinidin-3-O-Hexoside
8	753.16614	C36H32O18	+ H	1	Positive	8.00	18.00			Gallocatechin-Cianidin-3-O-Hexoside
9	463.12349	C22H22O11	+ H	1	Positive	5.00	35.00			Peonidin-3-O-galattoside
10	595.16575	C27H30O15	+ H	1	Positive	5.00	35.00			Cyanidin-3-O-rutinoside
11										

Figure S2: retention time (min.) of anthocyanins identified in PJs.

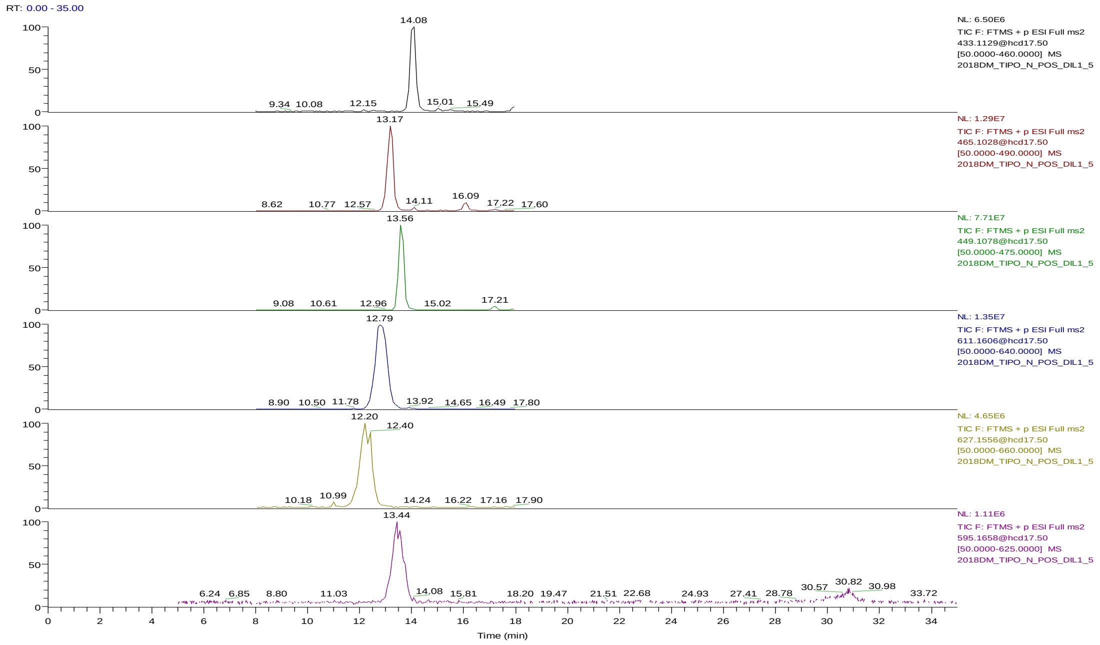

Figure S3: Inclusion list of phenolic acids , flavonoids and tannins with molecular formula and accurate mass of anthocyanins in negative ionization, by UHPLC–Orbitrap-MS.

Method editor — Inclusion List

File Edit Help Done

	Mass [m/z]	Formula [M]	Species	CS [z]	Polarity	Start [min]	End [min]	(N)CE	MSX ID	Comment
1	169.01425	C7H6O5	- H	1	Negative	8.00	25.00			Gallic Acid
2	329.08781	C14H18O9	- H	1	Negative	8.00	25.00			Vanillic Acid Hexoside
3	355.10346	C16H20O9	- H	1	Negative	8.00	25.00			Ferulic Acid Hexoside
4	433.04125	C19H14O12	- H	1	Negative	8.00	25.00			Ellagic Acid Pentoside
5	435.12967	C21H24O10	- H	1	Negative	8.00	25.00			Phlorizin
6	447.05690	C20H16O12	- H	1	Negative	8.00	25.00			Ellagic Acid Deoxy-Hexoside
7	447.09328	C21H20O11	- H	1	Negative	8.00	25.00			Kaempferol-3-O-Glucoside
8	463.08820	C21H20O12	- H	1	Negative	8.00	25.00			Quercetin-3-O-Hexoside
9	483.07803	C20H20O14	- H	1	Negative	8.00	25.00			Digalloil-Hexoside
10	609.14611	C27H30O16	- H	1	Negative	8.00	25.00			Rutin
11	633.07334	C27H22O18	- H	1	Negative	8.00	25.00			Corilagin
12	649.06825	C27H22O19	- H	1	Negative	8.00	25.00			Lagerstannin
13	783.06865	C34H24O22	- H	1	Negative	8.00	25.00			Pedunculagin
14	801.07921	C34H26O23	- H	1	Negative	8.00	25.00			Punigluconin
15	951.07452	C41H28O27	- H	1	Negative	8.00	25.00			Granatin B
* 16										

Figure S4: retention time (min.) of phenolic acids, flavonoids and tannins identified in PJs.

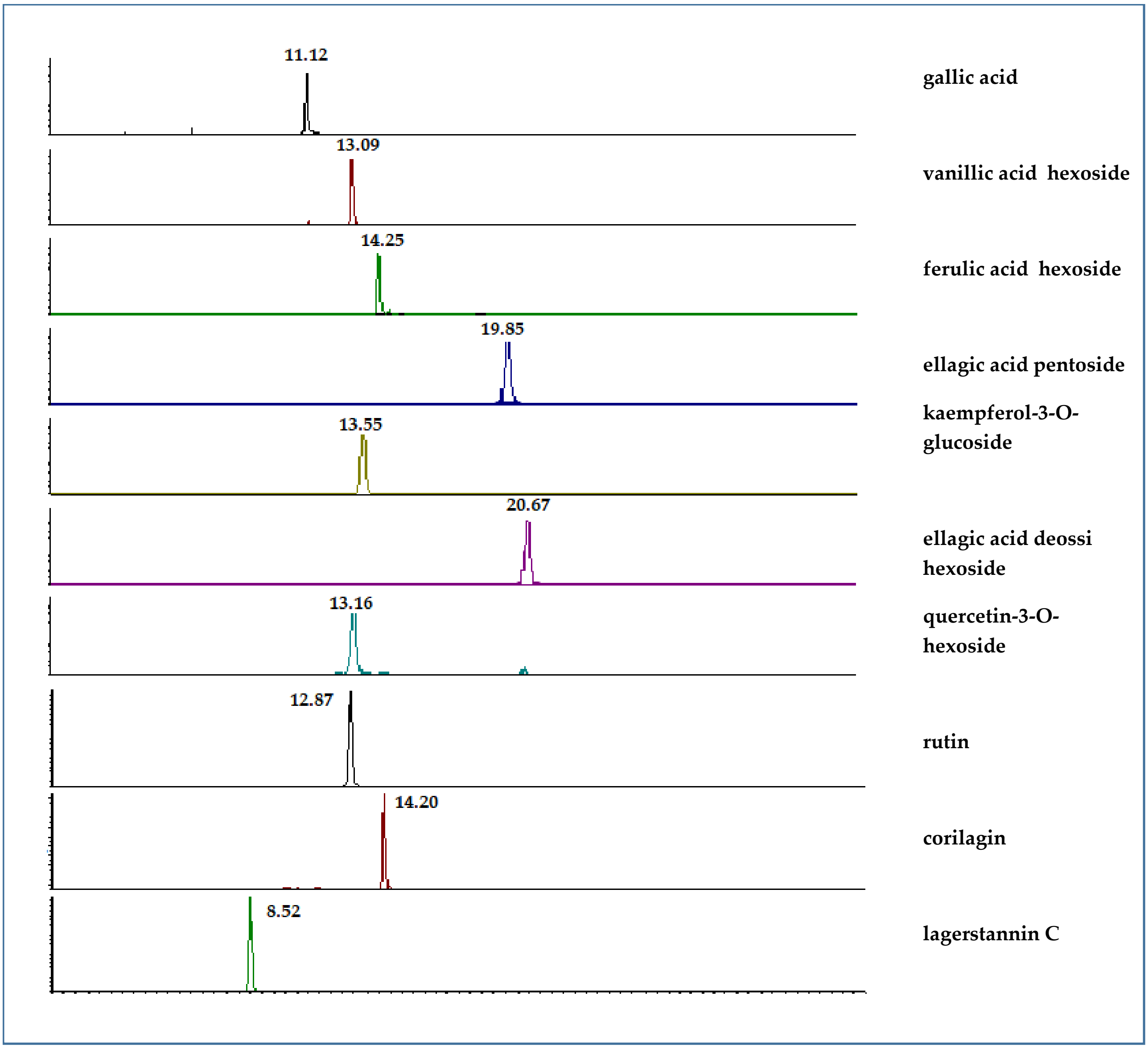

Supplement: Supplementary file 1 [file foods-09-01161-s001.pdf]
